# Relationship between Maternal Stress and Neurobehavioral Indicators of Preterm Infants in the Neonatal Intensive Care Unit

**DOI:** 10.3390/children11080889

**Published:** 2024-07-24

**Authors:** Bruna Abreu Ramos, Cibelle Kayenne Martins Roberto Formiga, Nayara Rodrigues Gomes de Oliveira, Patricia Gonçalves Evangelista Marçal, Rui Gilberto Ferreira, Tárik Kassem Saidah, Waldemar Naves do Amaral

**Affiliations:** 1 Department of Health Sciences, Federal University of Goiás (UFG), Goiania 74605-050, Brazil; bruna.ramos@hcb.org.br; 2 Department of Physical Therapy, State University of Goiás (UEG), Goiania 74075-110, Brazil; 3 Cibelle Kayenne Martins Roberto Formiga, Universidade Estadual de Goiás, Unidade ESEFFEGO, Avenida Oeste, Setor Aeroporto, Goiania 74075-110, Brazil; 4 Department of Science Applied to Health Products, State University of Goiás (UEG), Goiania 74075-110, Brazil; nayrgomes@ufg.br; 5 Department of Studies and Research, Federal University of Goiás (UFG), Goiania 74605-050, Brazil; 6 Department of Gynecology, Federal University of Goiás (UFG), Goiania 74605-050, Brazil

**Keywords:** child development, neonatal behavior, prematurity, psychological stress, parental stress

## Abstract

Background: Preterm birth and prolonged neonatal hospitalization are potential sources of stress for mothers of preterm and low birth weight infants. Aim: To evaluate maternal stress and its association with neurobehavioral indicators of preterm infants during hospitalization in the neonatal intensive care unit. Methods: A cross-sectional study was conducted in a neonatal intensive care unit of a hospital in Goiânia, Brazil. The study included preterm and low birth weight infants of both genders and their mothers. The Parental Stressor Scale: Neonatal Intensive Care Unit and the Neurobehavioral Assessment of the Preterm Infant were respectively applied to mothers and infants in the neonatal intensive care unit. Results: The study involved 165 premature infants and their mothers. The mean age of the mothers was 26.3 years and most had a high school education level (57.6%). Mothers perceived the experience of having an infant in the neonatal intensive care unit as moderately stressful (2.96 ± 0.81). The parental role alteration (4.11 ± 1.03) and sights and sounds (2.15 ± 0.90) subscales exhibited the highest and lowest stress levels, respectively. Significant correlations (rho < −0.3; *p* < 0.05) were found between maternal stress and neurobehavioral indicators of infants. In the multivariate analysis, low leg tone was a predictor of higher maternal stress. Low tone and limited arm movement were predictors of higher maternal stress in the maternal role item. Conclusions: The experience of having a preterm infant hospitalized was considered moderately stressful for mothers. Maternal stress levels were significantly correlated with low scores on neonatal neurobehavioral indicators.

## 1. Introduction

Preterm birth is one of the primary risk factors for developmental alterations in infants, affecting millions of families worldwide [1]. These risks can be of biological or environmental origin, such as the lower the birth weight and gestational age, the greater the risk of respiratory problems, feeding difficulties, neurological impairment, prolonged hospitalization in neonatal intensive care units (NICUs), negative developmental outcomes, and quality of life for the infant [2,3].

Despite the investment in technological resources and professional training for the care of these high-risk infants and the decrease in neonatal mortality rates, the impact of neonatal interventions on the physical and mental health of infants and their families needs to be analyzed [4]. The hospital environment is a source of stress for the infant due to rigid routines, numerous procedures, noise, constant lighting, monitoring equipment, and professionals handling the infant [5,6].

The family experiences moments of uncertainty regarding the future of the newborn and their own future and needs to confront new demands and adaptations, since admission to the NICU impacts quality of life in different aspects, such as personal, financial, occupational, and social [7]. These conditions also contribute to stressful experiences for parents, which may lead to sleep disturbances, excessive fatigue, and symptoms of anxiety and depression. The impact is particularly significant for mothers, whose emotional state may be affected by the experience of seeing their fragile infant struggling in the early stage of life [8,9].

Studies assessing parental stress in NICUs have provided valuable insights into the emotional challenge of mothers during the hospitalization of their preterm infants. These investigations highlight the complexity and intensity of experiences encountered by parents, particularly mothers, while navigating the treatment and care of their infant in the NICU [10,11].

The assessment of neurobehavioral indicators in preterm infants during their hospitalization period may predict cognitive and motor development outcomes in the early years of life [3,12]. Alongside other clinical risk indicators, neonatal neurobehavioral assessments may help identify infants at higher risk of developmental delay and provide opportunities for families to receive early intervention and targeted therapy [3,13].

As parents face their child’s hospitalization, anxiety increases regarding their baby’s unpredictable health status and uncertain future, resulting in psychological and emotional stress, as well as feelings of confusion and helplessness [10]. This experience can lead to a loss of confidence in parenting. Furthermore, the affectionate relationship between a premature baby and the mother can be challenging due to the baby’s instability, the NICU environment, and hospital policies [14]. Identifying the main stressors for mothers in this situation is crucial. A set of practices can be implemented to welcome and support these mothers, allowing them to feel essential to their preterm infants, face new stressors with more resilience, and neutralize the potential negative effects of early stress on the child’s development and health [15,16].

Therefore, studying preterm birth and maternal stress is essential in improving the health outcomes of preterm infants and supporting the emotional well-being of families affected by this challenging experience [7]. Promoting psychosocial support for mothers during hospitalization of their infants in the NICU may impact the emotional health of both, strengthening family bonds and promoting a healthier and more resilient life for preterm infants.

Stress responses among mothers of preterm infants experiencing the NICU environment have been widely reported. However, there is still a gap regarding the relationship between maternal stress and behavioral and neurodevelopmental changes in this preterm infant still in the NICU. Our hypothesis is that mothers, in addition to realizing the severity of the neonatal situation, are also vulnerable to the hospitalization process, and may be positively or negatively impacted by the behavioral aspects of neonates.

This study aimed to assess maternal stress perception and its relationship with neurobehavioral indicators of preterm infants during hospitalization in the NICU.

## 2. Methods

### 2.1. Study Design and Setting

A cross-sectional study was conducted from September 2018 to May 2021. The research was carried out at a public tertiary-level maternal and childcare hospital in the city of Goiânia, located in the central-west region of Brazil.

### 2.2. Participants

The eligible sample was mothers and infants admitted to the NICU. The infants of both genders were preterm (<37 weeks gestational age) and had low birth weight (<2500 g). In the case of twin or triplet pregnancy, only the firstborn was considered in the sample. Infants with chromosomal abnormalities, congenital disabilities, or hemodynamic instability during the neurobehavioral assessment were excluded.

The sample size was calculated using GPower software (version 3.2.1). The ideal sample size for this research was 164 children, based on an effect size of 0.25, an alpha value of 5%, and a statistical power of 0.95. Initially, 251 preterm infants were eligible for the study; however, infants had to be up to 37 weeks post-conceptional age for the neurobehavior to be assessed. Therefore, 86 infants were excluded due to not meeting the age eligibility for the instrument application. The final sample consisted of 165 preterm infants and their mothers.

### 2.3. Instruments and Outcomes

All eligible mothers and their infants were invited to participate in the study during the data collection period. Charts were reviewed to investigate maternal and infant data, such as gestational history, medication use, pregnancy complications, exam results, type of delivery, gestational age, birth weight, and length of hospital stay.

The socioeconomic status of mothers was classified using the Economic Classification Criterion of the Brazilian Association of Research Companies. This instrument considers the education level of the head of the household and the presence of household items (e.g., television, radio, bathroom, car, washing machine, and live-in maid). Its score ranges from 0 to 100 and divides the Brazilian population into six socioeconomic strata: Class A (45–100), Class B1 (38 to 44), Class B2 (29 to 37), Class C1 (23 to 28), Class C2 (17 to 22), and Class D-E (0 to 16).

The Parental Stressor Scale: Neonatal Intensive Care Unit (PSS:NICU) was applied to assess maternal parental stress in the NICU [17]. The PSS:NICU was developed in the United States and has been used in different countries [18,19,20]. It consists of 26 items distributed across three subscales that can be applied through an interview or self-administered format: “sights and sounds”, “infant behavior and appearance”, and “parental role alteration”. The scoring is based on a Likert-type scale ranging from 1 to 5: (1) not stressful, (2) slightly stressful, (3) moderately stressful, (4) very stressful, and (5) extremely stressful. The assessment also includes a statement that evaluates the overall stress level experienced by parents.

The present study used the PSS:NICU version adapted and validated to Brazilian Portuguese [21]. The overall stress level and responses of mothers to the 26 items distributed across the 3 subscales were considered in this study. The instrument was applied in an interview format that lasted 20 min.

The Neurobehavioral Assessment of the Preterm Infant (NAPI) was used to assess the neurobehavioral indicators in infants. The NAPI contains 71 items that measure the neurobehavioral performance of preterm infants between 32 and 37 weeks post-conceptional age (i.e., gestational age plus chronological age) [22]. The performance in each domain ranges from 0 to 100, and higher scores indicate better neurobehavioral development. Early risk of abnormal neurobehavioral development was considered when scores were one standard deviation below the mean. The validity and sensitivity of the NAPI were described using the Neonatal Medical Index, and inter-rater reliability ranged from 0.67 to 0.97 (sensitivity of 75% and specificity of 69%) [23]. The application time was 20 to 25 min.

Given the application time of the NAPI and the clinical conditions of infants hospitalized in the NICU, only six items were selected to achieve the objectives of this study: initial behavioral state, response to the scarf sign, leg and forearm resistance, popliteal angle, and power of active movements in arms and legs. These items were selected without the need to remove the infant from the incubator. A trained physiotherapist administered the NAPI, and all scores were recorded after a resting period of at least 30 min (i.e., no manipulation or invasive procedures before applying the NAPI). All infant assessments were performed by a single physiotherapist trained (BAR) and experienced in managing premature infants in the NICU.

### 2.4. Ethical Considerations

The study was approved by the research ethics committee of the Federal University of Goias (CAAE: 91780618.4.0000.8058) according to the Declaration of Helsinki and the National Health Council of Brazil.

### 2.5. Data Analysis

The mean ± standard deviation, median, minimum, and maximum values were calculated for continuous variables, whereas absolute and relative frequencies were calculated for discrete variables. Normality of data was assessed using the Kolmogorov–Smirnov test, recommended for samples larger than 50 participants. Spearman’s correlation test assessed the correlations between maternal stress items and neonatal neurobehavioral indicators. Subsequently, a multivariate analysis was conducted between cases of mothers with high stress levels (moderately, very, and extremely stressful), according to the total and subscale scores of the PSS:NICU, and the six neurobehavioral indicators of the NAPI. Data were analyzed using the Statistical Package for the Social Sciences 25.0 software (IBM Corp, New York, NY, USA), with a significance level set at 0.05.

## 3. Results

A total of 165 mothers and their infants were included. Mothers had a mean age of 26.9 years, high school education level (57.6%), cesarean delivery (63.0%), planned pregnancy (43.6%), were single (55.8%), and presented complications during pregnancy (87.9%). In terms of neonatal data, most were females (52.1%) with gestational age > 32 weeks (54.5%) and low birth weight (60.6%). Among the neonatal complications, respiratory distress syndrome (86.7%), infection (67.9%), use of invasive ventilation (41.8%), and oxygen therapy with other devices (71.5%) can be highlighted (Table 1).

The subscale with the lowest stress level was “sights and sounds”. Mean responses ranged from 1.26 to 3.3, and the following situations were the most stressful, according to parents: “having a machine (respirator) breathe for my baby” (mean of 3.3) and “the sudden noise of monitor alarms” (mean of 2.6). In the “infant behavior and appearance” subscale (mean of 2.8), the mean responses ranged from 1.38 to 4.1, with the most stressful items being “when my baby seemed to be in pain” (mean of 4.1) and “when my baby seemed sad” (mean of 3.7).

The subscale with the highest stress level was “parental role alteration” (mean of 4.1). In this subscale, mean responses ranged from 3.6 to 4.5, and the items “feeling helpless and unable to protect my baby from pain and painful procedures” (mean = 4.5) and “feeling helpless about how to help my baby during this time” (mean = 4.2) were considered very stressful. When analyzing maternal stress according to the Parental Stressors Scale (PSS), the total scale score was 2.9, indicating moderate stress (Table 2).

The results of the neurobehavioral assessment using the items of the NAPI scale are presented in Table 3. The category related to power of active movements (arms and legs) obtained the best response, while the poorest response was observed in the scarf sign, which evaluates upper limb tone. Additionally, the lower the scores in the initial behavioral state related to sleep during the assessments, the poorer the infant performance during the test. The mean score of 4.2 corresponded to inactive alertness, indicating that the children spent less time in sleep or drowsy states during the NAPI assessment.

Table 4 demonstrates the correlation coefficients between PSS:NICU and NAPI items. The higher the level of stress reported by mothers in the item “other sick babies in the room”, the lower the performance in leg resistance. Stress levels in the items “jerky and restless movements of my baby” and “feeling helpless and unable to protect the baby from pain and painful procedures” were correlated with performance in leg resistance and scarf sign. The higher the stress level in the item “not feeding my baby myself”, the higher the performance in the power of active movements of the legs. Similarly, the higher the score in the item “not having time to be alone with my baby”, the higher the performance in leg resistance and power of active movements of the legs.

The neurobehavioral indicators acting as predictors for maternal stress were the popliteal angle, scarf sign, and power of active movements of arms (Table 5).

## 4. Discussion

The study investigated the associations between maternal stress perception and neurobehavioral indicators of preterm and low birth weight infants admitted to a NICU in Brazil. Maternal stress was considered moderate according to the PSS:NICU. The highest and lowest scores were observed in the “parental role alteration” and “sights and sounds” subscales, respectively. Neurobehavioral indicators showed that infants exhibited a developmental level compatible with their post-conceptional age, were in a state of inactive alertness, and actively moved their arms and legs.

Parental stress assessment tools allow healthcare professionals from neonatal units to identify stress-promoting sources influencing parental responses and implement family-centered care practices [7]. In the present study, mothers were allowed to enter the hospital environment at specific times and observe some procedures performed on the infant. After the intubation, mothers could touch and hold the infant, and professionals guided them on how to adjust the positioning of tubes and catheters.

The parent–infant separation imposed by the NICU environment is widely discussed as one of the main sources of parental stress. Mothers often have to waive caregiving responsibilities during hospitalization and share them with nurses, doctors, and other healthcare professionals [16]. Mothers may also experience conflicts in the face of the infant’s vulnerability and feelings of deprivation regarding their maternal role while observing the healthcare professionals with their infants. On the other hand, the infant may often struggle to accept and recognize the maternal figure [24].

The parental–infant bond, the infant development, and adaptive and emotional behaviors may be negatively affected by the context and parental emotional reactions, such as fear, sadness, insecurity, and guilt [8,15]. Other stressful events that may disrupt parental bonding are prolonged hospitalization and lack of contact imposed by the NICU environment [25]. A cohort study indicated that parents involved with conventional care of preterm infants in the NICU showed higher levels of dissatisfaction and stress in the parental role subscale than those included in a family-centered care program [26]. In a study conducted in a NICU in the northern region of Brazil, mothers involved in infant care effectively assumed their maternal role and felt more competent in caring for the child [27].

Similar to our findings, Kegler et al. [9] observed that the “parental role alteration” was the subscale with the highest scores. This subscale is more evident in mothers who are unable to breastfeed, change diapers, bathe, hug, and cradle the infant as often as desired [27,28]. Consequently, mothers may feel frustrated and have difficulty recognizing the infant, which impacts the mother–infant bond and infant development [29].

A significant correlation between maternal stress and neurobehavioral indicators was observed. In the multivariate analysis, we observed that leg tone (analyzed by the popliteal angle) predicted maternal stress. Conversely, low tone and limited arm movement were predictors of stress in the parental role alteration item. These findings indicate that mothers perceive the state of their infant and behavior. Although mothers may not understand the risks and procedures performed by the NICU team, they seem to understand that the limited movement of the infant in the incubator and the presence of hypotonia indicate reduced vitality, including strength, vigor, and energy to move the body [12,30].

The neurobehavioral assessment conducted during the neonatal period before reaching full-term age may be a valuable predictor of developmental issues in follow-up services for preterm and low-birth-weight infants [3]. The levels of alertness and vigor exhibited by the infant during the neonatal period may reflect the maturation and adaptive responses to the extrauterine and hospital environments. Neurobehavioral disturbances may also impact infant development and relationships with parents and family [12].

In our study, despite the weak correlations, leg recoil and the power of active movements of the arm and leg were significantly correlated with some items of the maternal stress assessment. Neurobehavioral assessment during the neonatal period provides the first opportunity to understand the interactions between infants and the NICU environment [31]. In this context, the NAPI was developed to assess the neurobehavioral development of preterm infants before reaching full term and has the potential to evaluate neurobehavioral indicators during a period of vulnerability for the infant. Identifying associations between these risk factors and infant development provides effective support and efficient interventions targeted at the mother and family to improve infant development, especially in those preterm [10,32].

Studies in different countries have also found a low stress level in the “sights and sounds” subscale, such as the United States (mean of 2.3), Spain (mean of 2.2), and Australia (mean of 2.3) [18,19,20]. Parents from a NICU in Australia reported the need for invasive ventilation and the sudden noise of monitor alarms as the most stressful situations in the “sights and sounds” subscale [32]. In light of these results, the sights and sounds of machines and equipment in the NICU may have influenced the moderate stress observed in the “parental role alteration” subscale and impaired interactions between parents and infants [9,33].

A study by Yang et al. [11] was conducted to develop an understanding of mothers’ experiences breastfeeding a hospitalized preterm infant and the support needed to establish a milk supply during the period of separation from their infants. Mothers of preterm infants reported physically and mentally challenging breastfeeding experiences during the period they were separated from their newborns. They viewed expressing breast milk as integral to their maternal role, even though some found expressing breastmilk exhausting. With little professional support available, the mothers depended upon nonprofessionals to establish breastfeeding.

Regarding factors contributing to parental stress in the NICU, Smith et al. [13] emphasized the importance of social support and access to mental health resources to mitigate the negative impacts of parental stress. The study highlighted the need for interventions addressing not only the clinical needs of preterm infants but also the emotional well-being of parents and observed that parental support was crucial for promoting positive outcomes for the family. Furthermore, a literature review conducted by Oliveira et al. [34] analyzed studies on parental stress in NICUs and demonstrated the prevalence and adverse effects of parental stress on the mental health of parents and their ability to engage in the care of preterm infants. The review emphasized the importance of family-centered care approaches that recognize and address the emotional needs of parents, thus, promoting a more positive experience during the hospitalization of the infant in the NICU.

In the hospital environment, family care and psychological support for mothers are essential for coping with the situation of preterm birth and hospitalization, reducing anxiety, stress, and symptoms of depression [15]. After discharge, infants need to be followed up in an interdisciplinary outpatient clinic for preterm infants to help identify those at high risk for developmental problems, intervene with therapies and prophylaxis, and provide guidance to mothers on infant development and educational practices to facilitate bonds [4,35].

A study assessing the influence of preterm birth and maternal emotional health on infant development using the Bayley III scale demonstrated that mothers of preterm infants exhibited significantly higher levels of postpartum anxiety and depression compared with mothers of full-term infants. Although preterm infants showed poor development, changes in maternal emotional health were not associated with changes in infant development. The authors also observed risks for developmental delay when mothers of full-term infants experienced stress in four out of five assessed areas. Given the impact of emotional health and preterm birth on infant development, mothers of preterm and full-term infants should be monitored during the first year of life [36].

The present study has limitations regarding the design and data collection related to maternal outcomes. First, a cross-sectional study cannot establish a causal relationship between infant behavior and maternal stress. Additionally, maternal stress was assessed using indirect self-reported questionnaires. Despite these aspects, our findings indicate the need to implement strategies to minimize maternal distress and strengthen parental roles in the NICU. A trusting relationship between mothers and healthcare professionals may promote security and tranquility, minimize difficulties during hospitalization, favor mother–infant bonding, and increase family engagement in post-discharge follow-up programs. A recent systematic review of 46 studies on parenting interventions in preterm infants revealed that there is a wide range of intervention programs used to reduce parental stress. Despite this, most of the studies highlighted the need for greater clarification of the parental educational component [37]. Future studies involving neonatal intervention actions by NICU professionals are recommended to minimize stressful situations perceived by mothers, particularly those involving alterations in parental roles. Additionally, environmental organization and family support are advised to prevent or minimize motor developmental delay in preterm infants.

The study highlights the necessity of healthcare-team training for a more humane experience in the NICU, enabling positive engagement with mothers to address issues and emotional responses arising from hospitalization of their infants. This approach aims to facilitate the adaptation of mothers to the new roles and promote optimal development for the infant during and after hospitalization.

## 5. Conclusions

Mothers perceived the experience of having a preterm infant in the NICU as moderately stressful, particularly regarding the maternal social role. Despite weak correlations, an association between maternal stress and neurobehavioral indicators of infant tone and movement was identified, especially in the arms. These findings may assist professionals in conducting family-centered interventions in neonatal units, offering support and assistance to mothers of preterm infants, strengthening maternal emotional health, and enhancing mother–infant bonding during hospitalization.

## Figures and Tables

**Table 1:** Characteristics of mothers and preterm infants (*n* = 165).

Characteristics	Values
Maternal age (years)—mean ± SD	26.9 ± 6.8
Maternal education level	*n* (%)
College	37 (22.4)
High school	95 (57.6)
Elementary school	33 (20.0)
Socioeconomic status	
A1, B1, B2	80 (48.5)
C1, C2, D–E	85 (51.5)
City of residence	
Goiania (capital)	93 (56.4)
Countryside	72 (43.6)
Marital status	
Single	92 (55.8)
Married	73 (44.2)
Type of delivery	
Vaginal	61 (37.0)
Cesarean	104 (63.0)
Previous pregnancies	
One to three children	135 (81.8)
More than three children	30 (18.2)
Current pregnancy	
Planned pregnancy	72 (43.6)
Multiple pregnancy	19 (11.5)
Complications during pregnancy	145 (87.9)
High blood pressure	49 (29.7)
Infection	78 (47.3)
Bleeding	54 (32.7)
Diabetes	6 (3.6)
Number of obstetric ultrasounds—mean ± SD	4.88 ± 2.6
Infant gender	*n* (%)
Female	86 (52.1)
Male	79 (47.9)
Gestational age at birth	
≤32 weeks	75 (45.5)
>32 weeks	90 (54.5)
Weight at birth	
<1500 g	100 (60.6)
≥1500 g	65 (39.4)
Neonatal complications	
Respiratory distress syndrome	143 (86.7)
Infection	112 (67.9)
Apnea	38 (23.0)
Blood transfusion	19 (11.5)
Use of invasive ventilation	69 (41.8)
Use of oxygen therapy	118 (71.5)
Exogenous surfactant therapy	53 (32.1)
Phototherapy	110 (66.7)
Use of antibiotic therapy	103 (62.4)

*n*: Absolute frequency; %: Relative frequency; SD: Standard deviation.

**Table 2:** Maternal stress level according to the Parental Stressor Scale: Neonatal Intensive Care Unit.

Scale Items	Mean ± SD
Sights and sounds	2.15 ± 0.90
Presence of monitors and equipment	1.89 ± 1.32
Constant noises of monitors and equipment	2.26 ± 1.35
Sudden noises of monitor alarms	2.74 ± 1.49
Other sick babies in the room	1.47 ± 1.14
Large number of people working in the unit	1.26 ± 0.78
Having a machine (respirator) breathe for my baby	3.32 ± 1.75
Infant behavior and appearance	2.81 ± 1.08
Tubes and equipment on or near my baby	2.67 ± 1.64
Bruises, cuts, or incisions on my baby	3.14 ± 1.62
Unusual color of my baby	2.68 ± 1.57
Abnormal breathing patterns of my baby	3.22 ± 157
Small size of my baby	2.00 ± 1.42
Wrinkled appearance of my baby	1.38 ± 0.89
Seeing needles and tubes put in my baby	3.73 ± 1.43
Seeing my baby being fed by an intravenous line or tubes	2.90 ± 1.58
When my baby seemed to be in pain	4.10 ± 1.17
When my baby seemed sad	3.73 ± 1.50
The limp and weak appearance of my baby	2.53 ± 1.65
Jerky and restless movements of my baby	2.53 ± 1.51
My baby not being able to cry like other babies	3.04 ± 1.72
Parental role alteration	4.11 ± 1.03
Being separated from my baby	4.26 ± 1.21
Not feeding my baby myself	4.22 ± 1.30
Not being able to care for my baby myself	3.75 ± 1.63
Not being able to hold my baby when I want	3.92 ± 1.48
Feeling helpless and unable to protect my baby from pain and painful procedures	4.53 ± 0.99
Feeling helpless about how to help my baby during this time	4.29 ± 1.26
Not having time to be alone with my baby	3.62 ± 1.51
Total score	2.96 ± 0.81

SD: Standard deviation.

**Table 3:** Indicators of Neurobehavioral Assessment of the Preterm Infant.

Infant Data	Mean ± SD	Minimum	Maximum
Post-conceptional age (weeks)	34.31 ± 1.70	31.14	37.71
Peripheral oxygen saturation (%)	97.21 ± 2.60	90	100
Heart rate (bpm)	147.89 ± 19.28	100	197
Respiratory rate (breaths per minute)	50.56 ± 3.90	39	61
Weight at assessment (grams)	1712.62 ± 389.32	965	2690
Initial behavioral state	4.22 ± 0.69	3	6
Scarf sign	60.82 ± 15.17	0	100
Leg resistance			
Best score	3.38 ± 0.61	2	5
Mean score	2.99 ± 0.52	2	4
Forearm resistance			
Best score	3.28 ± 0.58	2	4
Mean score	2.75 ± 0.49	2	4
Popliteal angle	65.67 ± 19.97	33.3	100
Power of active movements			
Arms	75.16 ± 19.0	33.3	100
Legs	79.20 ± 19.24	33.3	100

SD = Standard deviation.

**Table 4:** Correlation between maternal stress and neurobehavioral indicators.

Maternal Stress (PSS:NICU)	NAPI	Spearman’s Correlation	*p*-Value
Other sick babies in the room	Leg resistance	−0.158	0.04
	Power of active movements—legs	−0.152	0.05
Having a machine (respirator) breathe for my baby	Scarf sign	−0.215	0.06
Small size of my baby	Scarf sign	−0.145	0.06
Jerky and restless movements of my baby	Scarf sign	−0.293	<0.001
Not feeding my baby myself	Power of active movements—legs	0.155	0.04
Feeling helpless and unable to protect my baby from pain and painful procedures	Scarf sign	−0.157	0.04
Not having time to be alone with my baby	Leg resistance	0.162	0.03
	Power of active movements—legs	0.183	0.01
Total score	Scarf sign	−0.152	0.05

NAPI: Neurobehavioral Assessment of the Preterm Infant. PSS:NICU: Parental Stressor Scale: Neonatal Intensive Care Unit.

**Table 5:** Multivariate analysis of neonatal neurobehavioral predictors for mothers experiencing moderate, high, and extremely high stress.

Variable	Mean ±SD	OR (95% CI)	*p*-Value	OR Adjusted (95% CI)	*p*-Value
Total stress score					
Popliteal angle	67.3 ±20.23	1.01 (0.99–1.03)	0.122	1.02 (1.00–1.04)	0.03
Parental role alteration					
Scarf sign	59.57 ±16.3	0.94 (0.89–0.99)	0.031	0.95 (0.89–1.00)	0.04
Power of active movements—arms	73.42 ±19.58	0.98 (0.95–1.01)	0.175	0.92 (0.84–1.00)	0.05

OR: Odds ratio; CI: Confidence interval; Multivariate analysis (*p* < 0.05).

## Data Availability

The raw data supporting the conclusions of this article will be made available by the authors on request.
